# New Technique of Double-Layer Alveolar Ridge Preservation Using Collagen Matrix on Periodontally Collapsed Extraction Region: Proof-of-Concept Case Study

**DOI:** 10.3390/jcm14113617

**Published:** 2025-05-22

**Authors:** Yu-Jin Kim, Jong-Bin Lee

**Affiliations:** 1 Department of Periodontology, Seoul Metropolitan Government–Seoul National University Boramae Medical Center, Seoul 07061, Republic of Korea; riseshine2@gmail.com; 2 Department of Periodontology and Research Institute of Oral Sciences, College of Dentistry, Gangneung-Wonju National University, Gangneung 25457, Republic of Korea

**Keywords:** alveolar ridge preservation, collagen matrix, dental implant, dental materials, tissue regeneration

## Abstract

**Background:** Alveolar ridge preservation (ARP) is widely used in clinical practice to prevent horizontal and vertical bone loss following tooth extraction. Conventional ARP uses a single coverage material with bone graft materials on a simple tooth extraction site. The objective of this study was to evaluate the clinical efficacy of a new double-layer ARP technique that additionally covers a collagen matrix at the top position, especially on the periodontally collapsed region following tooth extraction. **Methods:** In a clinical study process comparing our newly attempted ARP with the widely used conventional ARP, we discovered the clinical efficacy of our new ARP for specially selected cases. Because the extraction socket wall had collapsed and the entire ridge needed to be reconstructed, this procedure should be described as alveolar ridge augmentation. **Results:** Additional coverage of the collagen matrix protected the internal bone grafting and promoted external soft tissue regeneration and healing in sample cases. **Conclusions:** In conclusion, our procedure promotes the new generation of hard and soft tissues. It is particularly effective in regions requiring flapped surgery, such as areas with periodontal disease, long-span areas requiring multiple tooth extractions, and areas in which there is wide destruction of hard and soft tissues. Through this proof-of-concept case study, we aimed to standardize and evaluate this unprecedented surgical technique.

## 1. Introduction

After tooth extraction, the width and height of the remaining alveolar ridge in the extraction socket area inevitably change [1]. The alveolar process is a tooth-dependent structure, and the resorption of alveolar bone occurs due to the loss of the bundle bone surrounding the teeth. Notably, within three months post-extraction, an average of 50% of the buccal alveolar bone is absorbed, and the alveolar crest shifts toward the palatal/lingual side [2]. Such changes in the alveolar ridge can lead to the placement of implants in less-than-ideal positions, resulting in non-esthetic implant prosthetics and potential complications. Clinicians, therefore, must have a thorough understanding of the biological changes that occur following tooth extraction and must consider the regeneration of both hard and soft tissues at the extraction site [3]. In response to these challenges, numerous recent clinical studies have explored various methods and prognoses related to alveolar ridge preservation (ARP), aimed at mitigating the inevitable volume loss of the alveolar ridge after extraction [4].

ARP has demonstrated promising short-term outcomes in preventing both horizontal and vertical bone loss post-extraction. Conventional ARP involves using bone graft material along with a covering membrane. In cases of minimal periodontal bone loss, such as single-tooth simple extraction sockets, the application of a membrane and suturing with flapless surgery is straightforward, making it an easily performed clinical procedure [5]. However, in extraction sockets compromised by periodontal disease or multiple tooth extractions (i.e., long-span region), various challenges arise [6], including difficulties in membrane application and maintenance. In such cases, if a resorbable membrane becomes widely exposed in the oral cavity, it may degrade quickly. This directly leads to barrier function loss and insufficient space maintenance. As a result, it causes inadequate bone regeneration and a lack of new bone formation, as well as increases the risk of bacterial contamination and infection [7,8,9,10,11,12]. Consequently, primary closure is often attempted [13], and in some instances, a double-membrane technique is employed [5].

If ARP is applied in the challenging situations mentioned above, it is often insufficient to completely compensate for the severe soft tissue deficiency due to the excessive alveolar bone contraction, and additional soft tissue surgery may be required. Instead of using autologous tissues such as free gingival graft (FGG) or connective tissue graft (CTG), recent studies have increasingly focused on utilizing xenogenic collagen matrix as soft tissue substitutes. The xenogenic collagen matrix used in our study is composed of two layers: the upper compact layer, which protects the bone graft during secondary healing and facilitates suturing; and the lower porous layer, which stabilizes the blood clot and promotes early vascularization and cell growth, thereby accelerating soft tissue healing [14].

Despite the advancements in this field, no recent studies have explored the additional use of soft tissue substitutes in ridge preservation or augmentation procedures. The aim of this study was to evaluate the clinical efficacy of a new ARP technique that incorporates an additional layer of xenogenic collagen matrix on the uppermost part of the treatment area (i.e., new double-layer ARP technique). This innovative approach has shown promise in enhancing soft tissue regeneration and secondary healing accompanied by bone regeneration, particularly in periodontally compromised extraction sites. After analyzing the accumulated data from various ARPs, we aim to standardize and evaluate our new double-layer ARP technique through this proof-of-concept case study based on specifically selected cases.

## 2. Materials and Methods

All the patients described in this study were treated by one periodontal specialist (i.e., JBL) with same surgical protocol in the Department of Periodontology. We provided all patients with a sufficient explanation about the surgery and performed the surgery after confirming the patient’s consent. In patients with collapsed extraction sockets showing hard and soft tissue deficiency after the removal of teeth, a soft tissue substitute (i.e., xenogenic collagen matrix) was applied in conjunction with conventional ARP to preserve and enhance both the hard and soft tissues at the implant placement sites. We present a sample case of this new double-layer ARP technique based on the successful data from our clinical cases. This will serve as a technical note for suggesting a surgical protocol for a future clinical study.

### 2.1. Case 1 (Single Tooth, Severe Buccal Bone Loss, and Cystic Lesion on the Extraction Region)

**Figure 1:**
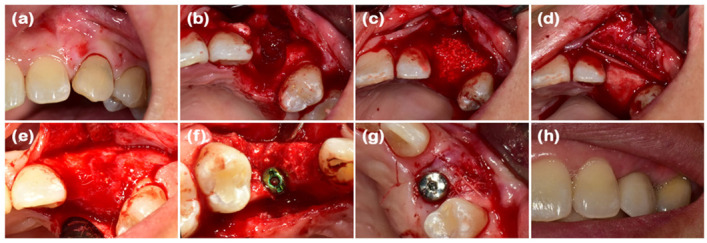
Clinical photographs of case 1. (**a**) The initial presentation of the maxillary left canine prior to extraction. (**b**) The buccal bone defect is exposed after full-thickness flap elevation. (**c**) The defect is filled with deproteinized bovine bone mineral (DBBM). (**d**) The bone graft area is covered with soft-type resorbable collagen membrane, hard-type resorbable collagen membrane on the buccal side, and finally, collagen matrix in the outer position. (**e**) Full-thickness flap elevation reveals adequate bone volume. (**f**) Implants are placed in the sufficiently augmented bone area. (**g**) The second implant surgery is performed. (**h**) Definitive prosthesis connection.

**Figure 2:**
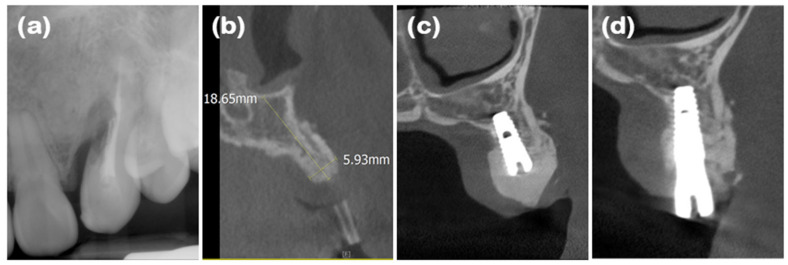
Radiographs of case 1. (**a**) Periapical radiograph showing external root resorption of maxillary left canine. (**b**) Cone-beam computed tomography (CBCT) image displaying augmented site after healing. Alveolar ridge width was measured at approximately 5.93 mm, which is sufficient for placement of standard diameter implant. (**c**) Post-implant placement with guided bone regeneration, demonstrating bone material surrounding implant fixture. (**d**) Following second implant surgery, contour of bone graft was well maintained.

A 44-year-old female patient was referred from a private dental clinic with the chief complaint of external root resorption in the upper left canine. She had no significant medical history, and upon initial examination, a 6 mm periodontal pocket was observed on the mesial and buccal sides of the maxillary left canine (#23). Clinical and radiographic examination revealed external resorption on the mesial aspect of the root (Figure 1a and Figure 2a). Extraction of #23 and the new double-layer ARP technique were planned.

After performing full-mouth scaling, #23 was extracted and ARP was performed a month later. A horizontal incision was made on the palatal side, and a full-thickness flap, including the interdental papilla, was elevated buccally. The periapical cystic lesion was removed, revealing extensive horizontal and vertical bone defects as well as buccal bone plate loss (Figure 1b). A synthetic bone graft material mixed with collagen (Osteon 3 collagen^®^ (biphasic calcium phosphate with collagen), Genoss Co., Ltd., Suwon, Republic of Korea) was inserted into the extraction socket, and to fully cover the alveolar crest, a resorbable collagen membrane with soft-type stiffness (Collagen membrane 2^®^, Genoss Co., Ltd.) was applied. To reconstruct the buccal bone wall, a resorbable collagen membrane with medium-type stiffness (Collagen membrane P^®^, Genoss Co., Ltd.) was placed over the buccal side of the socket. Due to the extensive buccal bone loss, a soft tissue graft between the membrane and the flap was necessary to compensate for soft tissue collapse and delayed healing. A xenogenic collagen matrix (Collagen graft 2^®^, Genoss Co., Ltd.) was inserted buccally and slightly on the crestal area (Figure 1c,d). The area was sutured without tension using 6-0 Vicryl (Ethicon, INC., a Johnson & Johnson company, Somerville, MA, USA) and 5-0 Black nylon (AILEE Co., Ltd., Busan, Republic of Korea), and intentional secondary healing (open healing) was induced. Upon suture removal 10 days later, secondary healing was observed, with no significant findings other than partial exposure of the bone graft material on the buccal side. The secondary healing area was continuously monitored. After confirming the epithelialization of the soft tissue and the complete regeneration of the interdental papilla between the adjacent teeth, the first implant surgery was performed 9 months after the alveolar ridge augmentation. Prior to implant placement, the width and height of the remaining alveolar ridge were observed to be in good condition (Figure 1e and Figure 2b). A full-thickness flap was elevated, revealing a sufficiently augmented alveolar bone with a width of approximately 6 mm bucco-palatally. The implant was placed in an ideal position (Superline Φ3.5 × 10 mm, Dentium, Seoul, Republic of Korea), and to compensate for the loss of bone graft material due to drilling, additional guided bone regeneration (GBR; Osteon 3 collagen^®^ and Collagen membrane P^®^, Genoss Co., Ltd.) was performed on the palatal side (Figure 1f and Figure 2c). Five months after the first implant surgery, the second implant surgery was carried out (Figure 1g and Figure 2d). Seven months after the first implant surgery, a customized titanium abutment and zirconia crown were placed with screw-type connection. The interdental papilla between #22 and #23 was regenerated, and 4 mm of buccal keratinized mucosa was stably observed (Figure 1h).

### 2.2. Case 2 (Multiple Posterior Teeth, Periodontitis, and Bucco-Palatal Collapsed Extraction Region)

A 48-year-old male patient presented with discomfort in the upper right molar region. He had no significant medical history, and clinical and radiographic examinations revealed bone loss extending to the apex of the maxillary right first molar (#16) and advanced periodontitis in the second molar (#17). Accordingly, extraction of #16 and #17 and the new double-layer ARP technique were planned.

**Figure 3:**
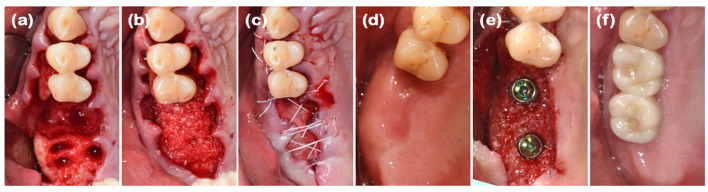
Clinical photographs of case 2. (**a**) A severe buccal bone defect observed on the maxillary right molar following full-thickness flap elevation. (**b**) The defect filled with DBBM. (**c**) Resorbable collagen membrane placed over the bone graft, with a collagen matrix layered on the outer position. The operative area was left slightly open with hidden-x and simple interrupted sutures. (**d**) The pre-operative view before implant placement. (**e**) The implants were placed in the sufficiently augmented bone area. (**f**) Definitive prosthesis connection.

**Figure 4:**
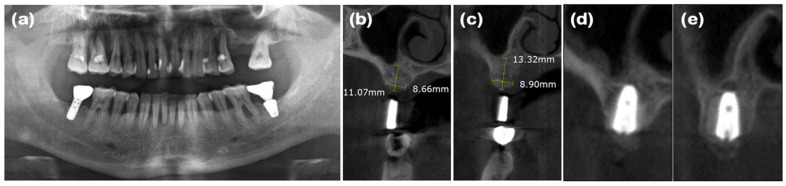
Radiographs of case 2. (**a**) The pre-operative panoramic view taken during the initial visit. (**b**,**c**) CBCT images displaying the augmented site after healing. The alveolar ridge width was measured at approximately 8.66 and 8.90 mm on the maxillary right first and second molar areas, respectively, which is sufficient for the placement of a standard diameter implant. (**d**,**e**) Implants were placed in the maxillary right first and second molar areas, respectively.

First, full-mouth scaling was performed. After the extraction of #16 and #17, a full-thickness flap was elevated, revealing buccal and palatal bone loss at the #16 site (Figure 3a and Figure 4a). Xenograft mixed with collagen (Bio-Oss collagen^®^ (deproteinized bovine bone mineral with collagen), Geistlich Parma AG, Wolhusen, Switzerland) was applied to the extraction sites of #16 and #17, and a resorbable collagen membrane (Bio-Gide compressed^®^, Geistlich Parma AG) was placed over the graft (Figure 3b). The incision was extended due to the posterior flap design, which increased flap elevation, and there was significant alveolar bone loss and soft tissue deficiency at the #16 extraction site. Even with secondary healing, it was anticipated that the newly regenerated soft tissue would be thin and insufficient. Therefore, along with ARP, a xenogenic collagen matrix (Mucograft^®^, Geistlich Parma AG) was applied. Hidden-X sutures [15] and simple interrupted tension-free sutures were placed using 4-0 Biotex (Purgo Biologics, Seongnam, Republic of Korea), and secondary healing was intended (Figure 3c). Upon suture removal 10 days later, secondary healing was confirmed, and the patient was monitored. Ten months later, a full-thickness flap was elevated for implant placement, and an augmented alveolar bone width of 8 mm was observed (Figure 3d and Figure 4b,c). The implant was placed in the ideal position (Superline Φ5.0 × 10 mm, Dentium), and GBR (Bio-Oss^®^ and Bio-Gide compressed^®^, Geistlich Parma AG) was performed to compensate for the graft material lost during drilling (Figure 3e and Figure 4d,e). Five months after the first implant surgery, the second implant surgery was performed, and the final prosthesis—consisting of a customized titanium abutment and zirconia crown with screw-type connection—was placed seven months after the first implant surgery (Figure 3f). Clinical examination revealed stable horizontally and vertically augmented alveolar bone and 6 mm of keratinized mucosa around the implant on the buccal side.

### 2.3. Case 3 (Multiple Anterior Teeth, Periodontitis, and Labio-Lingual Collapsed Extraction Region)

A 69-year-old male patient presented with a chief complaint of mandibular anterior tooth extraction and implant placement. He had no significant medical history. Clinical examination revealed crowding in the mandibular anterior region, and radiographic examination showed bone loss extending to the apices of the teeth (#31, #32, #41, and #42). The new double-layer ARP technique was planned after the extraction of the mandibular anterior teeth.

**Figure 5:**
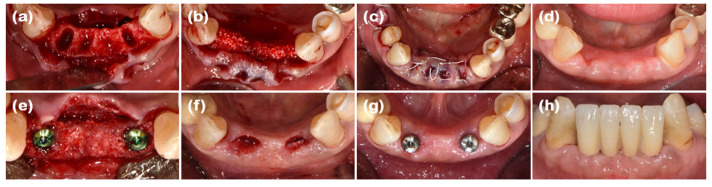
Clinical photographs of case 3. (**a**) A severe buccal bone defect observed on the mandibular anterior area following full-thickness flap elevation. (**b**) The defect was filled with DBBM. (**c**) Resorbable collagen membrane was placed over the bone graft, with a collagen matrix layered on the outer position. The operative area was left slightly open with tension-free sutures. (**d**) The pre-operative view before implant placement. (**e**) Implants were placed in the sufficiently augmented bone area. (**f**) A U-shaped incision in the mandibular right and left lateral incisors for healing abutment connection. (**g**) Two weeks after the second implant surgery, showing regenerated interdental papilla around both implant areas. (**h**) Definitive prosthesis connection.

**Figure 6:**
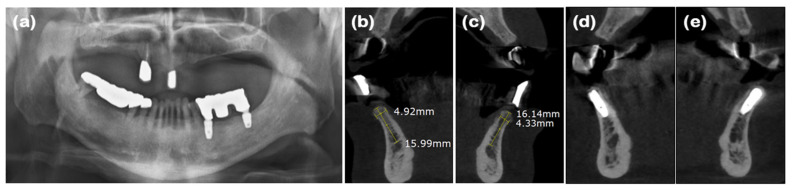
Radiographs of case 3. (**a**) The pre-operative panoramic view taken during the initial visit. (**b**,**c**) CBCT images displaying the augmented site after healing. The alveolar ridge width was measured at approximately 4.92 and 4.33 mm on the mandibular right and left lateral incisor areas, respectively, which is sufficient for the placement of a standard diameter implant. (**d**,**e**) The implants were placed in the mandibular right and left lateral incisor areas, respectively.

After performing full-mouth scaling, four mandibular anterior teeth were extracted gently. To preserve the interdental papilla after extraction, an incision was made on the lingual side, and the interdental papilla without cutting was included in the full-thickness flap, which was elevated buccally. Buccal bone plate loss was observed (Figure 5a and Figure 6a). Synthetic bone graft material mixed with collagen (Osteon 3 collagen^®^, Genoss Co., Ltd.) was applied to the extraction site (Figure 5b), and a resorbable collagen membrane (Collagen membrane P^®^, Genoss Co., Ltd.) was placed over the bone graft. To promote soft tissue regeneration through secondary healing, a xenogenic collagen matrix (Collagen graft^®^, Genoss Co., Ltd.) was placed over the resorbable collagen membrane and tension-free sutures were applied (Figure 5c). During the healing process, the shape and form of the alveolar bone and interdental papilla, as well as their harmony with the adjacent teeth, were maintained (Figure 5d and Figure 6b,c). Three months after the ARP, the first implant surgery was performed in the #32 and #42 regions (Implantium Φ3.8 × 10 mm, Dentium) (Figure 5e and Figure 6d,e). A horizontal incision was made slightly on the lingual side, and a full-thickness flap was elevated, revealing 4 mm of augmented bone width. The implant was placed in the ideal position. Every two to three months after the first implant surgery, the preserved shape of the interdental papilla was continuously observed. The second implant surgery was performed 3 months after the first implant surgery, and the final prosthesis consist of a customized titanium abutment and zirconia bridge with cementation-type connection were placed 4.5 months after the first implant surgery (Figure 5f–h). Ultimately, the interdental papilla harmonized with the adjacent teeth, and 4 mm of buccal keratinized mucosa was stably observed.

### 2.4. Case 4 (Multiple Posterior Implants, Peri-Implantitis, and Collapsed Ridge of Implant Removal Region)

A 67-year-old female patient presented with inflammation and bleeding around the implants in the right mandibular second premolar and first molar (#45 and 46) areas, which had been placed over 10 years ago in a private dental clinic. Her medical history included hyperthyroidism and degenerative arthritis, but these were well controlled. Initial examination revealed 7–8 mm periodontal pockets in the #45 and 46 implant areas and less than 1 mm width of buccal keratinized mucosa. Radiographic examination showed severe alveolar bone loss exceeding half the length of the implant fixtures. According to Decker’s classification [16,17], the case was diagnosed as unfavorable, and implant removal was planned. In addition, extensive ARP (i.e., new double-layer ARP technique) for hard and soft tissue reconstruction, followed by re-implantation, was planned.

**Figure 7:**
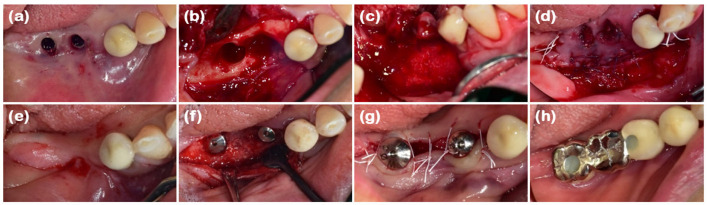
Clinical photographs of case 4. (**a**) Deficiency of keratinized tissue observed on the buccal side of the mandibular right implant removal areas following the removal of the implant prosthesis. (**b**) A severe bone defect exposed after buccally positioned incision and full-thickness flap elevation. (**c**) The defect was filled with DBBM. (**d**) Resorbable collagen membrane was placed over the bone graft, with a collagen matrix layered on the outer position. The surgical area was covered using an apically positioned flap combined with the modified Eldan–Mejchar technique. (**e**) The pre-operative view before implant placement. (**f**) The implants placed in the sufficiently augmented bone area. (**g**) The second implant surgery was performed. (**h**) Definitive prosthesis connection.

**Figure 8:**
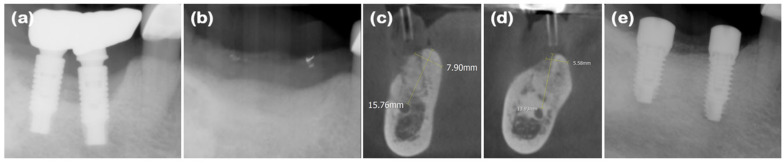
Radiographs of case 4. (**a**) The pre-operative periapical radiograph. (**b**) The post-operative periapical radiograph of the mandibular right molar areas, demonstrating the area filled with bone graft material. (**c**,**d**) CBCT images displaying the augmented site after healing. The alveolar ridge width was measured at approximately 7.90 and 5.58 mm on the mandibular first and second molar areas, respectively, which is sufficient for the placement of a standard diameter implant. (**e**) The periapical radiograph after the second implant surgery.

After full-mouth scaling, the #45 and #46 implant prosthesis was removed, and the implants were extracted in a step-by-step process (Figure 7a and Figure 8a). Buccal horizontal bone loss was observed, and bone graft material (Bio-Oss collagen^®^, Geistlich Parma AG) along with a resorbable collagen membrane (Bio-Gide compressed^®^, Geistlich Parma AG) was applied to the implant removal sites (Figure 7b,c). To secure the vestibular depth, the modified Edlan–Mejchar technique was performed (Figure 7d). A xenogenic collagen matrix (Mucograft^®^, Geistlich Parma AG) was applied to the exposed periosteal area to promote keratinized mucosa regeneration in the buccal vestibule and the ARP site, and the area was sutured without tension using 4-0 Biotex (Purgo Biologics), and 5-0 and 6-0 Vicryl (Ethicon, INC., a Johnson & Johnson company). Clinical follow-up was conducted monthly after the ARP surgery, and after 2 months, an augmented alveolar ridge, keratinized mucosal tissue formation, and deepened vestibular depth were confirmed (Figure 7e and Figure 8b–d). Five months after the ARP surgery, a full-thickness flap was elevated for implant placement in the #45 and #47 areas, revealing the augmented alveolar bone, and the first implant surgery was performed (#45 implant: USII SA Φ4.5 × 10 mm; #47 implant: Φ5.0 × 8.5 mm; Osstem Co., Seoul, Republic of Korea) (Figure 7f). Additional GBR using Bio-Oss^®^ and Bio-Gide^®^ (Geistlich Parma AG) was performed on the buccal side of the fixtures and sutured; the sutures were removed after 10 days with no significant side-effects observed. Five months after the first implant surgery, healing abutments were connected, and the implant stability quotient (ISQ) for the #45 implant level was 90 and for the #47 implant level was 93, with increased keratinized mucosa observed (Figure 7g and Figure 8e). Seven months after the first implant surgery, a customized titanium abutment and porcelain-fused-to-metal crown were placed with a screw-type connection. Around the prosthesis, a stable band of keratinized mucosa approximately 3 mm in width was observed (Figure 7h).

### 2.5. Clinical and Radiographic Evaluation

In anterior cases, an esthetic evaluation of the implant prosthesis was performed after the placement of the final prostheses. After 6 to 12 months from the final prosthesis delivery, the esthetic evaluation was conducted on the clinical recall-check using the pink esthetic score and the white esthetic score (PES/WES) [18]. These measurements were each performed at least three times by two different dentists who did not perform the surgery, and the final values were decided upon thorough in-depth discussion among the measurers. Before implant surgery, measurements of the alveolar bone were conducted using the CBCT image program (INFINITT Co., Ltd., Phillipsburg, NJ, USA), and after the final prosthesis was delivered, the periodontal specialist (i.e., JBL) evaluated the keratinized mucosa in a clinical examination (INFINITT Co., Ltd., Phillipsburg, NJ, USA). All implants were placed at 1 mm subcrestal level for zero-bone-loss concepts, and accordingly, ridge width (i.e., bucco-palatal) was measured based on the length of implant fixture at 2 mm below the level of the ridge crest. In addition, the buccal keratinized mucosa width was measured around the implant prosthesis using a periodontal probe (PCP12 probe; Hu-Friedy Manufacturing, Co., LLC, Chicago, IL, USA). These two measurements were made by the periodontal specialist (i.e., JBL) who performed the surgery to eliminate inter-measurer errors.

## 3. Results

In the four cases presented above, ARP was performed on areas with defects caused by severe periodontal lesions, and a collagen matrix was additionally applied to promote hard and soft tissue regeneration. In two anterior cases (case 1 and case 3), an esthetic evaluation of the implant prosthesis was performed after the placement of the final prostheses (Table 1 and Table 2). For all four cases, the outcomes of ARP were assessed by summarizing the ridge width observed using cone-beam computed tomography (CBCT) and the keratinized mucosa width measured through clinical examination (Table 3).

In the PES/WES evaluation for case 1, the PES was 7 points, with a total of 3 points deducted each due to a color difference and the gingival margin being positioned more coronally than the adjacent teeth. The WES for the prosthesis was 7 points because the shape was not identical to the contralateral tooth, and the color appeared more muted than that of the adjacent teeth (Table 1). In case 3, the PES was 8 points due to slight deficiencies in the formation of the mesiodistal interdental papilla. The WES was also 8 points, with a 1-point deduction each for tooth shape and form, considering harmony with the adjacent teeth (Table 2).

In all cases, sufficient bone length and width was achieved after ARP to allow for the placement of a standard implant, and more than 2 mm of keratinized mucosa was secured around the implant (Table 3).

## 4. Discussion

Recent studies have shown that ARP can be successfully performed without primary closure when resorbable collagen membranes are used appropriately. Additionally, many studies have reported successful outcomes in terms of the complete exposure of the collagen membrane after ARP. Some authors used a single layer of a resorbable collagen membrane, while others used double-layers for open healing ARP [5]. However, collagen membranes tend to degrade when exposed to the oral cavity, and there are concerns that they may not provide sufficient stabilization for bone grafts during the healing period [7,8,9,10,11,12], particularly when used with single coverage rather than double coverage [5,19,20]. For example, Garcia et al. [21] conducted a systematic review and meta-analysis demonstrating that sites without membrane exposure achieved significantly greater horizontal bone gain (74%) and defect reduction (27%) compared to exposed sites. Elgali et al. [22] reported that membrane exposure negatively impacts the outcome of lateral ridge augmentation, primarily due to contamination and compromised wound stability.

In our study, instead of applying double-layers of a simple collagen membrane, we applied a collagen matrix over the upper part of the collagen membrane with a hybrid purpose. The inner layer of the collagen membrane preserved the underlying bone graft, while the outer layer of the collagen matrix contributed to soft tissue regeneration and favorable healing, as confirmed in the sample case. Several studies have reported the advantages of xenogenic collagen matrix. By avoiding donor site surgery, the procedure time is shorter, and it provides clinical outcomes similar to autografts. It also produces results that are esthetically and histologically similar to the autogenic mucosa and causes less pain, resulting in higher patient satisfaction [23].

There is a viewpoint that ARP, which involves intentional secondary healing (i.e., open healing), has limitations in regenerating both alveolar bone and soft tissue. Emphasizing the importance of primary closure, it is suggested that addressing the deficiency of soft tissue in the area where the tooth was present is necessary to regenerate functional bone and soft tissue suitable for implant placement. Some systematic reviews have also reported that ARP alone does not significantly increase keratinized tissue [24]. To cover the soft tissue on the upper part of the treatment area, Becker [25] introduced a method of coronally repositioning the buccal flap, while Rosenquist [26] presented a technique for covering the extraction socket with soft tissue using a buccal pedicle flap rotation procedure. Additionally, autologous tissue grafts, such as FGG or CTG, are often performed to increase the amount of keratinized mucosa around implants. FGG is a surgical method used to increase the width of keratinized mucosa, while CTG is a predictable surgical procedure for soft tissue augmentation, such as root coverage. However, these methods have several drawbacks, including postoperative discomfort for patients due to additional soft tissue surgery, a limited quantity of donor tissue, residual scars in the palate, and non-esthetic outcomes due to differing textures and colors at the graft site [27].

Alternative methods to surgery using autologous tissue have been reported. These alternatives include biomaterials such as acellular dermal matrix and xenogenic collagen matrix. Acellular dermal matrix is an allogeneic connective tissue graft that undergoes a decellularization process. However, it has been reported to show significant contraction after the healing period and does not fully integrate histologically [28]. Xenogenic collagen matrix is being studied as a substitute for autologous tissue grafts in procedures like FGG or CTG. It is an absorbable 3D matrix designed for soft tissue regeneration. Made from Type I and Type III collagen using a standardized manufacturing process without cross-linking or chemical treatments, it can be used for root coverage and the regeneration of keratinized mucosa [23]. However, research on the use of soft tissue substitutes for regeneration in ARP remains limited.

In this study, ARP was performed with flapped surgery in patients who required horizontal and vertical bone augmentation due to the absence of residual bone. A collagen matrix was used to compensate for ridge contraction and to increase the keratinized mucosa around the implants. In case 1, the buccal bone plate at site #23 had been resorbed, resulting in a narrow alveolar ridge width in the bucco-palatal direction, which was insufficient for implant placement. To augment the buccal bone, xenogenic bone graft material, two types of resorbable collagen membranes, and a xenogenic collagen matrix were applied, sequentially. At the time of implant placement, a ridge width of approximately 5.93 mm was observed bucco-palatally, and continuous, intact soft tissue regeneration, including preservation of the interdental papilla, was achieved. In case 2, the bone wall at extraction site #16 had resorbed, necessitating both bone and soft tissue augmentation for predictable implant placement. Periodontal flap surgery was also performed on the adjacent teeth, resulting in a wider incision. A xenogenic collagen matrix was applied to promote adequate soft tissue regeneration. At the time of implant placement, ridge widths of approximately 8.66 and 8.90 mm were observed bucco-palatally at sites #16 and #17, respectively, along with a continuous soft tissue contour. In case 3, following the extraction of teeth #31, #32, #41, and #42, the buccal bone plate had been resorbed. After broad ridge augmentation, a xenogenic collagen matrix was applied to aid in soft tissue regeneration and preservation of the interdental papilla. At the time of implant placement, ridge widths of approximately 4.92 and 4.33 mm were observed bucco-palatally at sites #32 and #42, respectively, along with continuous soft tissue contour. Upon completion of the implant prosthetics, a natural appearance of the interdental papilla was observed continuously. In case 4, horizontal and vertical alveolar bone defects were observed in explanted sites #46i and #47i, along with a shallow buccal vestibule and insufficient keratinized mucosa. A xenogenic collagen matrix was applied to the buccal and crestal side to ensure sufficient alveolar bone augmentation and keratinized mucosa for implant placement, and the modified Edlan–Mejchar technique was used to deepen the buccal vestibule. At the time of implant placement, ridge widths of approximately 7.90 and 5.58 mm were observed bucco-palatally at sites #45 and #47, respectively, while maintaining the natural soft tissue appearance and deepened vestibule. In terms of the anterior teeth in case 1 and case 3, the PES/WES values were measured as 7–8 points, confirming that although the periodontal defects were severe, good esthetic results could be obtained. In the cross-sectional CBCT views and clinical examinations of all cases, the alveolar bone width was sufficient to accommodate at least a narrow-diameter implant in the anterior region and at least a regular-diameter implant in the posterior region. In addition, the width of the buccal keratinized mucosa was confirmed to be consistently well maintained at 2 mm or more in all cases. Although there are many opposing views, it is important to secure the presence of keratinized mucosa with more than a 2 mm width around the implant for the health and maintenance of the implant [29].

In all cases, increases in alveolar bone and keratinized mucosa were observed and maintained stably during the follow-up period. There were no significant adverse effects associated with the use of the collagen matrix, and all cases showed favorable healing. When collagen matrix was applied as a soft tissue substitute in conjunction with ARP in cases where hard and soft tissue defects were anticipated after extraction, the following advantages were observed. In extraction sockets with severe horizontal and vertical buccal bone defects, bone and soft tissue regeneration was smooth and continuous, allowing for easy incision and suturing in the appropriate position during implant surgery. Additionally, in long-span areas with the loss of two or more teeth where keratinized mucosa was insufficient, more than 2 mm of keratinized mucosa could be secured around the implants, and preservation of the interdental papilla of adjacent teeth was possible.

Although there are many studies evaluating hard tissue after ARP, evaluations of soft tissue remain limited. In cases of periodontal compromised extraction sockets involving periodontal disease and multiple tooth extractions, considerations for soft tissue are more important than those for hard tissue. In long-span areas, the disadvantage of hard tissue can be overcome by adjusting the implant placement position, considering splinting after short implant placement, or suggesting other prosthetic alternatives. However, it has been reported that the lack of soft tissue causes difficulties from the beginning of the surgery involving incision and suturing, and is also a challenging and controversial factor in terms of follow-up maintenance after implant placement [30,31].

Our study has focused on this aspect. Based on the results of this proof-of-concept case study, we plan to use the new double-layer ARP technique on a large-scale clinical study comparing the conventional ARP technique, applying various collagen matrixes, and evaluating the sole application of collagen matrix in various collapsed extraction regions with severe hard and soft tissue loss. For this purpose, our study has introduced a protocol for the new surgical technique for specially selected sample cases. Therefore, to accurately verify the effectiveness of this technique, clinical, radiological, and histomorphological analyses and measurements of a large number of regenerated tissue samples are required in our planned further study. This study has value as a pilot case report for future research, and it is significant in identifying the advantages of the additional application of collagen matrix in conventional procedures. As mentioned above, this study has limitations in that it is a report on special cases, so a clinical study using a large number of subjects is needed. In addition, clinical, radiological, and histological investigations should be added to evaluate the inflammatory response to the material, qualitative/quantitative properties of the new soft tissue, and changes in the alveolar ridge over time. We will conduct these further studies to meet these conditions under a thorough plan.

## 5. Conclusions

Our new double-layer ARP technique, with additional collagen matrix coverage compared to the conventional ARP technique, enhances total tissue regeneration and healing in periodontally collapsed extraction regions.

## Figures and Tables

**Table 1:** Pink esthetic score and white esthetic score of case 1.

Pink Esthetic Score (PES)		White Esthetic Score (WES)	
Mesial papilla	2	Tooth form	1
Distal papilla	2	Tooth volume/outline	1
Curvature of facial mucosa	1	Color (hue/value)	2
Level of facial mucosa	1	Surface texture	2
Soft tissue color and texture	1	Translucency	1
**Total**	7		7

**Table 2:** Pink esthetic score and white esthetic score of case 3.

Pink Esthetic Score (PES)		White Esthetic Score (WES)	
Mesial papilla	1	Tooth form	1
Distal papilla	1	Tooth volume/outline	1
Curvature of facial mucosa	2	Color (hue/value)	2
Level of facial mucosa	2	Surface texture	2
Soft tissue color and texture	2	Translucency	2
**Total**	8		8

**Table 3:** Ridge width and keratinized mucosa width.

	Case 1	Case 2		Case 3		Case 4	
**Tooth position**	#23	#16	#17	#32	#42	#45	#47
**Ridge width (mm) ^1^**	5.93	8.66	8.90	4.92	4.33	7.90	5.58
**Keratinized mucosa width (mm) ^2^**	4	6	6	4	4	3	3

^1^ Bucco-palatal width on cone-beam computed tomography (CBCT) at 2 mm below the level of the ridge crest. ^2^ Buccal keratinized mucosa width around the implant prosthesis.

## Data Availability

The data supporting the results of this paper are entirely provided by the corresponding author. In addition, some of these data were used in part in a paper published in a Korean Journal (i.e., Journal of Dental Rehabilitation and Applied Science), and the editorial board of that journal has approved submission and publication for this journal. Data can be provided by request from the editor. The data used and/or analyzed during the current study are available from the corresponding author on reasonable request.
